# Development and implementation of a customised rapid syndromic diagnostic test for severe pneumonia

**DOI:** 10.12688/wellcomeopenres.17099.3

**Published:** 2022-10-12

**Authors:** Vilas Navapurkar, Josefin Bartholdson Scott, Mailis Maes, Thomas P Hellyer, Ellen Higginson, Sally Forrest, Joana Pereira-Dias, Surendra Parmar, Emma Heasman-Hunt, Petra Polgarova, Joanne Brown, Lissamma Titti, William PW Smith, Jonathan Scott, Anthony Rostron, Matthew Routledge, David Sapsford, M. Estée Török, Ronan McMullan, David A Enoch, Vanessa Wong, VAP-Rapid investigators, Martin D Curran, Nicholas M Brown, A John Simpson, Jurgen Herre, Gordon Dougan, Andrew Conway Morris

**Affiliations:** 1 John V Farman Intensive Care Unit, Cambridge University Hospitals NHS Foundation Trust, Cambridge, CB2 0QQ, UK; 2 Cambridge Institute of Therapeutic Immunology & Infectious Disease, University of Cambridge, Cambridge, CB2 0AW, UK; 3 Translational and Clinical Research Institute, University of Newcastle, Newcastle upon Tyne, NE2 4HH, UK; 4 Newcastle upon Tyne Hospitals NHS Foundation Trust, Newcastle upon Tyne, NE7 7DN, UK; 5 Clinical Microbiology and Public Health Laboratory, Cambridge University Hospitals NHS Foundation Trust, Cambridge, CB2 0QQ, UK; 6 School of Clinical Medicine, University of Cambridge, Cambridge, CB2 0QQ, UK; 7 Infectious Diseases, Cambridge University Hospitals NHS Foundation Trust, Cambridge, CB2 0QQ, UK; 8 Pharmacy Department, Cambridge University Hospitals NHS Foundation Trust, Cambridge, CB2 0QQ, UK; 9 Department of Medicine, University of Cambridge, Cambridge, CB2 0QQ, UK; 10 Microbiology, Cambridge University Hospitals NHS Foundation Trust, Cambridge, CB2 0QQ, UK; 11 Wellcome-Wolfson Institute for Experimental Medicine, Queen's University Belfast, Belfast, BT9 7BL, UK; 12 Respiratory Medicine, Cambridge University Hospitals NHS Foundation Trust, Cambridge, CB2 0QQ, UK; 13 Division of Anaesthesia, Department of Medicine, University of Cambridge, Cambridge, CB2 0QQ, UK

**Keywords:** Antimicrobial stewardship, Critical Care, Molecular pathology, Pneumonia

## Abstract

**Background:** The diagnosis of pneumonia has been hampered by a reliance on bacterial cultures which take several days to return a result, and are frequently negative. In critically ill patients this leads to the use of empiric, broad-spectrum antimicrobials and compromises good antimicrobial stewardship. The objective of this study was to establish the performance of a syndromic molecular diagnostic approach, using a custom TaqMan array card (TAC) covering 52 respiratory pathogens, and assess its impact on antimicrobial prescribing.

**Methods:** The TAC was validated against a retrospective multi-centre cohort of broncho-alveolar lavage samples. The TAC was assessed prospectively in patients undergoing investigation for suspected pneumonia, with a comparator cohort formed of patients investigated when the TAC laboratory team were unavailable.

Co-primary outcomes were sensitivity compared to conventional microbiology and, for the prospective study, time to result. Metagenomic sequencing was performed to validate findings in prospective samples. Antibiotic free days (AFD) were compared between the study cohort and comparator group.

**Results:** 128 stored samples were tested, with sensitivity of 97% (95% confidence interval (CI) 88-100%). Prospectively, 95 patients were tested by TAC, with 71 forming the comparator group. TAC returned results 51 hours (interquartile range 41-69 hours) faster than culture and with sensitivity of 92% (95% CI 83-98%) compared to conventional microbiology. 94% of organisms identified by sequencing were detected by TAC. There was a significant difference in the distribution of AFDs with more AFDs in the TAC group (p=0.02). TAC group were more likely to experience antimicrobial de-escalation (odds ratio 2.9 (95%1.5-5.5)).

**Conclusions:** Implementation of a syndromic molecular diagnostic approach to pneumonia led to faster results, with high sensitivity and impact on antibiotic prescribing.

## Introduction

For many decades the diagnosis of infectious diseases has relied on a combination of clinical assessment and microbiological culture. However, cultures are frequently negative ^1, 2^ and can take several days to return a result ^3^. Optimising antimicrobial therapy can be challenging, especially in patients who are at risk of multidrug resistant organisms ^2^. In critically ill patients, this frequently results in the empiric use of broad-spectrum agents, with predictable consequences for antimicrobial resistance and other forms of antimicrobial-related harm such as drug toxicity and disruption of the microbiome ^4^. Conversely, failure to identify the causative organism can lead to inappropriate antimicrobial therapy, which is associated with poor outcomes ^5^.

Pneumonia amongst intubated and mechanically ventilated, critically ill patients can be especially difficult to diagnose ^6^. Most critically ill patients are systemically inflamed ^7^, clinical examination is unreliable ^8^ and there are multiple causes of radiographic lung infiltrates, most of which are non-infectious ^9, 10^.

The development of host-based biomarkers for infection, such as C-reactive protein ^11^, procalcitonin ^12^, and alveolar cytokine concentrations ^10, 13 ^ have been advanced as useful measures to help rationalise antimicrobial use. However, their utility in the diagnosis ^11, 12^ and antimicrobial stewardship ^14, 15^ of pneumonia has been challenged.

There is, therefore, a pressing need for rapid, sensitive, multi-pathogen-focussed diagnostic tests for pneumonia ^16^. However, although intensive care physicians appreciate the potential advantages of such diagnostics, they are also wary of potential downsides ^17^, emphasising the need for evaluation of these tests in real-life clinical practice.

 TaqMan array cards (TAC) enable the conduct of multiple simultaneous single-plex real-time polymerase chain reaction (RT-PCR), with this format allowing rapid and straightforward customisation. This customisation allows for a wider range of organisms than those found in existing commercially available tests, and rapid modification to address emerging threats. Although TACs have shown promising performance relative to conventional microbiology ^18^, our previous experience demonstrated that a TAC with restricted coverage of common respiratory pathogens had a limited impact on clinical decision making in critically ill patients ^19^. We therefore set out to develop and implement a multi-pathogen array that would have broad applicability for severe pneumonia.

## Methods

### Ethical and regulatory approvals and funding

The prospective study was approved by the Leeds East Research Ethics Committee (17/YH/0286), Cambridge University Hospitals NHS Foundation Trust was the sponsor, and registered with clinicaltrials.gov (NCT03996330). The assessment of routinely collected data from the comparator group received a consent waiver as data came from routinely collected clinical data and was conducted under a protocol approved by the institutional review board (A095506). The protocol has been deposited on Zenodo ^20^. VAPrapid ^15^ was approved by the England and Northern Ireland (13/LO/065) and Scotland (13/SS/0074) National Research Ethics Service committees and sponsored by Newcastle upon Tyne Hospitals NHS Foundation Trust.

### Card development

**Figure 1.**
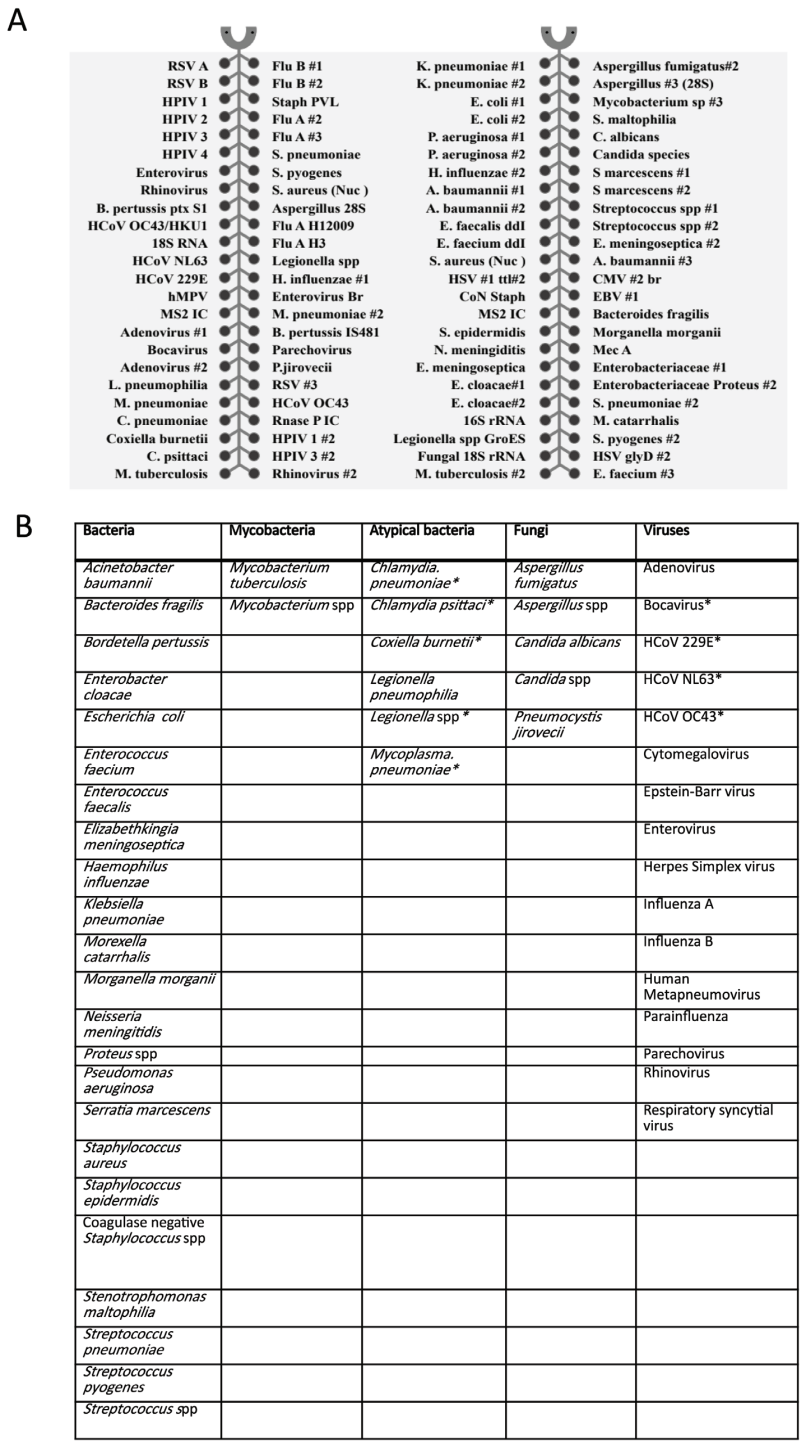
**A**) TAC layout and targets, **B**) summary of organisms covered. * indicates organisms not routinely tested for by conventional microbiology in Clinical Microbiology and Public Health Laboratory, Public Health England, Cambridge. HCoV -human coronavirus.

The local microbial ecology was reviewed using previous conventional microbiological culture data from the hospital. This was supplemented by review of the literature concerning causative organisms reported in ventilator-associated and community-acquired pneumonia and the authors’ previous experience of molecular diagnostics in pneumonia ^1, 2, 6, 13, 19, 21^. Species- or genus-specific primer/probe sequences were identified by reviewing the literature for well cited and fully validated real-time PCR assays with the presumption that, where possible, each organism should be covered by two sequences to minimise false positive results. In the absence of a published validated assay, one was designed in-house, normally targeting a housekeeping gene in the first instance (i.e. gyrB, rpoB, ssrA, dnaJ, recN) following the guidelines set out previously ^22^. Briefly, all assays were subjected to a comprehensive *in silico* analysis using BLAST analysis to ensure specificity of primers and probes and examined for possible cross-reactions with other high priority organisms. Potential for adverse probe/primer interactions and melting temperature (T _*m*_) were assessed using OligoAnalyzer 3.1. If necessary, sequences were modified accordingly to remove any cross reactions and to ensure T _*m*_ of 55-60°C, to allow for uniform amplification. The 52 organisms (23 bacteria, 2 mycobacteria, 6 atypical bacteria, 5 fungi and 16 viruses) covered by the card are shown in Figure 1. Sequences on the card have been deposited on Zenodo ^23^.

### TAC procedure

Nucleic acid extraction from clinical samples was undertaken using the NUCLISENS easyMAG platform (cat number 280140, Biomerieux, Marcy L’Etoile, France), in accordance with the manufacturer’s instructions. Nucleic acids were extracted from 500 µL of input sample, with a dilution of MS2 bacteriophage added pre-extraction to act as an internal extraction and inhibition control.

Cards were run on the QuantStudio 7 Flex platform (cat number 4485701,ThermoFisher, Waltham, MA, USA), following a modified version of the method previously described ^18^. Briefly, 50 µL of each nucleic acid extract was mixed with 50 µL of TaqMan Fast Virus 1-step mastermix (cat number 4444436, ThermoFisher) and 100 µL of RNase-free water, before 98 µL was added in 2 consecutive sample loading ports covering all 96 targets. Reverse transcriptase real-time PCR was undertaken according to the following amplification protocol: 50°C for five minutes, 95°C for 20 seconds, then 45 cycles of 95°C for one second followed by 60°C for 20 seconds. Detection of a clear exponential amplification curve with a cycle threshold (CT) value ≤38 for any single gene target was reported as a positive result for the relevant pathogen.

The study was undertaken prior to the coronavirus 2019 (COVID-19) pandemic.

### Card validation

***Technical validation*.** The card was initially validated against our large bank of DNA extracts from a diverse range of microorganisms, known positive/negative clinical specimens, and all available EQA panels from Quality Control for Molecular Diagnostics covering all targets except *C. burnetti*, *C. psittaci*, *C. pneumonia*, *M. pneumoniae* and *Elizabethkingia meningoseptica*. For *C. burnetti*, *C. psittaci*, *C. pneumonia* and *M. pneumoniae* control DNA extracts were purchased (Vircell, Granada, Spain, Catalogue numbers MBC018, MBC013, MBC011 and MBC035 respectively). A panel of nine synthetic control plasmids containing all our target sequences (with 20 nucleotides each side of the primer target sites also included) were generated ( GenScript, Leiden, NL) and used to quality check each batch of TAC plates and determine the limit of detection of each assay. As a demonstration of clinical utility, complete concordance was achieved against the five organisms from the Quality Control for Molecular Diagnostics 2018 Sepsis EQA Pilot Study ( *Streptococcus pneumoniae*, *Pseudomonas aeruginosa*, *Klebsiella pneumoniae*, *Enterococcus faecalis* and *Candida albicans* ( Table 1).

**Table 1.** Performance of TaqMan array card in the Public Health England Quality Control for Molecular Diagnostics 2018 Sepsis EQA Pilot Study. (TM: transport medium, ddl: D-alanine-D-alanine ligase).

Sample content	Matrix*	TaqMan array cards results (Ct Value)
*Streptococcus pneumoniae*	TM	S pneumoniae #1 29.602 S pneumoniae #2 28.624 Streptococcus spp #1 29.060 Streptococcus spp #2 29.027
*Pseudomonas aeruginosa*	TM	P aeruginosa #1 28.806 P aeruginosa #1 28.350
*Klebsiella pneumoniae*	TM	K pneumoniae #1 25.650 K pneumoniae #2 25.106 Enterobacteriaceae 26.922
*Enterococcus spp*	TM	E faecalis ddl 26.641
*Streptococcus pneumoniae*	Blood	S pneumoniae #1 31.353 S pneumoniae #2 30.811 Streptococcus spp #1 31.735 Streptococcus spp #2 31.952
*Candida albicans*	Blood	Candida albicans 34.313 Candida spp 33.124 Fungal 18S 30.839
*Pseudomonas aeruginosa*	Blood	P aeruginosa #1 28.065 P aeruginosa #1 30.647
*Klebsiella pneumoniae*	Blood	K pneumoniae #1 26.770 K pneumoniae #2 25.213 Enterobacteriaceae 26.715
*Enterococcus spp*	Blood	E faecalis ddl 30.431
Negative	Blood	PCR Negative

***Retrospective cohort validation*.** A retrospective cohort validation was conducted using stored bronchoalveolar lavage (BAL) samples obtained during the 24 centre VAPrapid trial of a biomarker for the diagnosis of ventilator-associated pneumonia ^15^. VAPrapid centres used semi-quantitative microbiological culture as the reference standard but did not undertake routine testing for viruses or *Pneumocystis jirovecii*. The stored samples were analysed on the TAC using methods outlined above.

***Prospective evaluation*.** The protocol for the prospective study, finalised prior to first recruitment, has been deposited on Zenodo ^20^. The prospective study was registered with clinicaltrials.gov ( NCT03996330; June 24, 2019).

Setting

Patients were recruited from a 20-bedded teaching hospital Intensive Care Unit (ICU). The unit is a mixed general medical-surgical unit which supports transplant and haematology-oncology services.

Recruitment

Between February 2018 and August 2019, patients were eligible for inclusion if they were receiving invasive mechanical ventilation, and if the treating intensive care specialist (consultant) suspected pneumonia and was planning to perform diagnostic bronchoscopy. Exclusions were lack of a proxy decision maker to provide study assent, and lack of laboratory study team availability to perform the TaqMan array card assay (the laboratory study team were routinely unavailable from Friday 5pm to Monday 8am, and also sporadically unavailable due to leave). Patients were included consecutively when the study team was available. Written informed consent was obtained when patients had capacity at time of enrolment. For those lacking capacity, written proxy assent (nominated or personal consultee advice) was obtained prior to study inclusion, and retrospective consent was sought if capacity was regained whilst the patient remained in hospital. Patients were identified by the treating team and included prospectively and consecutively when the laboratory study team were available.

Patients who were not included in the study because of a lack of TAC laboratory team availability, and those from the month prior and month following the study, formed the comparator group.

Sampling procedure

Bronchoscopy for both TAC and comparator patients was conducted in accordance with the unit protocol. Briefly, the scope was wedged in a sub-segment of the area with maximal radiographic change, or where frank pus was seen emerging. In diffuse infiltrates a sub-segment of the right middle lobe or lingula was selected. Saline in 50ml aliquots up to a volume of 200ml was instilled and withdrawn after a dwell time of 10 seconds. Where samples were taken out of hours (Monday-Thursday 5pm-8am and Sunday 8am-Monday 8am), samples were stored at 4°C prior to processing within 24 hours, in accordance with existing laboratory procedures.

TAC testing

The samples for TAC were processed as set out above. The TAC was run by a dedicated laboratory team who did not undertake the conventional PCR or cultures, with blinding also assured by the results of the TAC being obtained before those from conventional microbiology.

Conventional microbiological testing

BAL samples were processed according to the UK Standards for Microbiology Investigations (SMI) ^24^. Samples were inoculated onto a range of solid agars and incubated in both air and 5–10% CO _2_ targeting conventional respiratory tract pathogens, *Staphylococcus aureus*, Enterobacteriales and Pseudomonads. Any organism with growth >10 ^4^ CFU/mL was identified to species level using matrix-assisted laser desorption isonisation time of flight (MALDI TOF) mass spectrometry (Bruker MALDI Biotyper Sirus (IVD)Bruker Ltd, Coventry, UK). A scanty mixed growth with no predominant organism was reported as ‘mixed respiratory tract flora’ and not characterised any further. Extended culture was performed for Legionella, Nocardia, anaerobes, fungi and Mycobacterium species. Growth at <10 ^3^ CFU/ml was reported as negative.

A single in-house multiplex PCR assay formed the basis of conventional testing for common respiratory viruses (adenovirus, enterovirus, human metapneumovirus, influenza A virus, influenza B virus, parainfluenza virus, rhinovirus, and respiratory syncytial virus). In-house monoplex PCR assays were used for the detection of *Pneumocystis jirovecii*. *Aspergillus* spp. were tested for by culture on Sabouraud Dextrose Agar with chloramphenicol(cat number PO0161A, Thermofisher), with or without testing for the presence of galactomannan antigen in serum (serum GM) and BAL (BAL GM) by Platelia™ *Aspergillus* enzyme immunoassay (cat number 62794, Bio-Rad Laboratories, Hercules, CA). As well as routine culture, *Legionella pneumophila* serotype 1 was tested for by the detection of antigen in urine, using the Alere BinaxNOW™ Legionella Urinary Antigen Card (cat number 852-000, Abbott Rapid Diagnostics, Stockport, UK) with positive tests confirmed in the national reference laboratory. Conventional laboratory methods were not routinely available to detect coronaviruses.

As an experimental assay, the results of the TAC were not included in the laboratory information system, blinding the assessors of the reference standard to the TAC results.

Return of results to clinical team

Following review by a consultant clinical scientist, results were returned to the ICU team. Clinical microbiology advice was available 24 hours/day, and patients underwent weekday daily combined ICU-Microbiology multi-disciplinary reviews in keeping with existing unit practice (weekend microbiology input was available on request). The study did not mandate any course of action by the treating clinical team. Conventional microbiology results were returned to clinicians via the electronic health record; however, in practice these were returned after the TAC results.

Outcome measures

The co-primary outcome measures were sensitivity, using conventional microbiology as the reference standard and time to result compared to conventional microbial culture. Time to result for microbial culture was taken as time from completion of lavage to first organism identification, or confirmation of negative growth if no organisms were detected.

Secondary outcome measures were sensitivity compared to metagenomic microbial sequencing, time to result compared to conventional PCR, days alive and free of antibiotics (antibiotic-free days, AFDs) in seven and 28 days following lavage and change in antibiotic therapy in the seven days following lavage. Qualitative assessment of whether TAC results impacted on antimicrobial change was assessed by clinical notes review by a member of the study team who was not involved in the decision-making process (VW).

Statistical analysis

The difference in median time to result for conventional culture and TAC was assessed by Wilcoxon’s matched-pairs test. Where conventional PCR failed, or where the lab did not test for the organism, the corresponding tests from the TAC were removed from calculation of diagnostic performance. Indeterminate cultures (‘mixed upper respiratory tract flora’) were considered negative. A sensitivity analysis, coding failed conventional PCR and organisms not tested ‘negative’ was also undertaken. Comparisons of distribution of antibiotic free days between TAC and comparator groups was by Mann-Whitney U test, differences in proportions of escalation and de-escalation decisions were assessed by Chi ^2^ test. Analyses were conducted using Prism v9.1 (Graphpad Inc, La Jolla, CA).

Study size

A planned prospective study size of 100 patients evaluated by TAC was selected to balance cost against including sufficient numbers to be able to make a judgement on the card’s clinical utility. As the co-primary endpoint was time to result in a real-world setting that had not been previously evaluated, we did not undertake a formal power calculation.

Metagenomic sequencing

To further validate the results of the prospective TAC assay, metagenomic sequencing was undertaken. Residual BAL samples (average 40 mL) from 98 out of the 100 patients were used for metagenomic sequencing (two could not be sequenced due to presence of potential containment level 3 organisms and lack of a CL3 facility in the sequencing laboratory). BAL was centrifuged at 500 x g for five minutes to separate the host cells (pellet) from the bacterial, viral and fungal pathogens (supernatant). One mL of each sample supernatant was filtered through a 0.45 µm filter and used for viral RNA and DNA extraction using a QIAamp MinElute Virus Spin kit (Qiagen), using an on-column DNase step for viral RNA. Reverse transcription and random amplification of both viral DNA and cDNA was carried out as described previously ^25^. The remaining supernatant was centrifuged at 3220 x g for 30 minutes and the pellet was subjected to host cell depletion using MolYsis Basic5 (Molzym, Bremen, DE) followed by bacterial/fungal DNA extraction using a QIAamp DNA Mini kit (Qiagen, Hilden, DE). Half of the DNA was submitted for HiSeq 4000 shotgun metagenomic sequencing, while the other half was used to amplify the 16S V4 region using barcoded primers ^26^ ( The Earth Microbiome Project), amplicons were sequenced by Illumina MiSeq sequencing. All samples were sequenced at the Wellcome Sanger Institute, and raw read data are available at the European Nucleotide Archive (ENA) project PRJEB29011 with study accession numbers ERP111277, ERP111280, ERP112277, and ERP018622. Amplicon data were analysed using Qiime2 v2019.10.0 ^27^. Single-end sequences were denoised using Deblur ^28^, and classified using a feature classifier built from the Greengenes 13_8 99% OTUs taxonomy database. For metagenomic shotgun sequence data, human reads were first removed from the data using Bowtie2 v2.3.5 ^29^. 5’Human-depleted paired reads were then classified using Kraken2 v2.0.8 ^30^. For bacterial targets, a curated bacterial database based on the Genome Taxonomy Database ^31^ was used for classification. For viral and fungal pathogens, the standard Kraken2 viral and fungal databases were used. Qiime2 and Kraken2 tabular outputs were subsequently processed in R (version 3.5.3) ( R core team) to calculate the proportions of reads mapping to individual taxa for each sample.

The outputs for each of the sequencing approaches were compared to paired negative controls and analysed for the presence of fungal, viral and bacterial reads. Fungal and viral organisms were reported if they were the dominant species and/or the read counts were above the determined background levels. Bacterial organisms that were reported if identified by both shotgun and 16S amplicon sequencing, or present as the dominant species in shotgun sequencing above the background levels. Organisms that had low read counts, or were only identified by 16S but were detected by TAC, were reported as low confidence hits.

## Results

### Technical validation

Following initial validation against stored DNA extracts and synthetic plasmids, all microorganisms from the Quality Control for Molecular Diagnostics 2018 Sepsis EQA Pilot Study were successfully detected ( Table 1).

### Retrospective cohort validation

**Figure 2.**
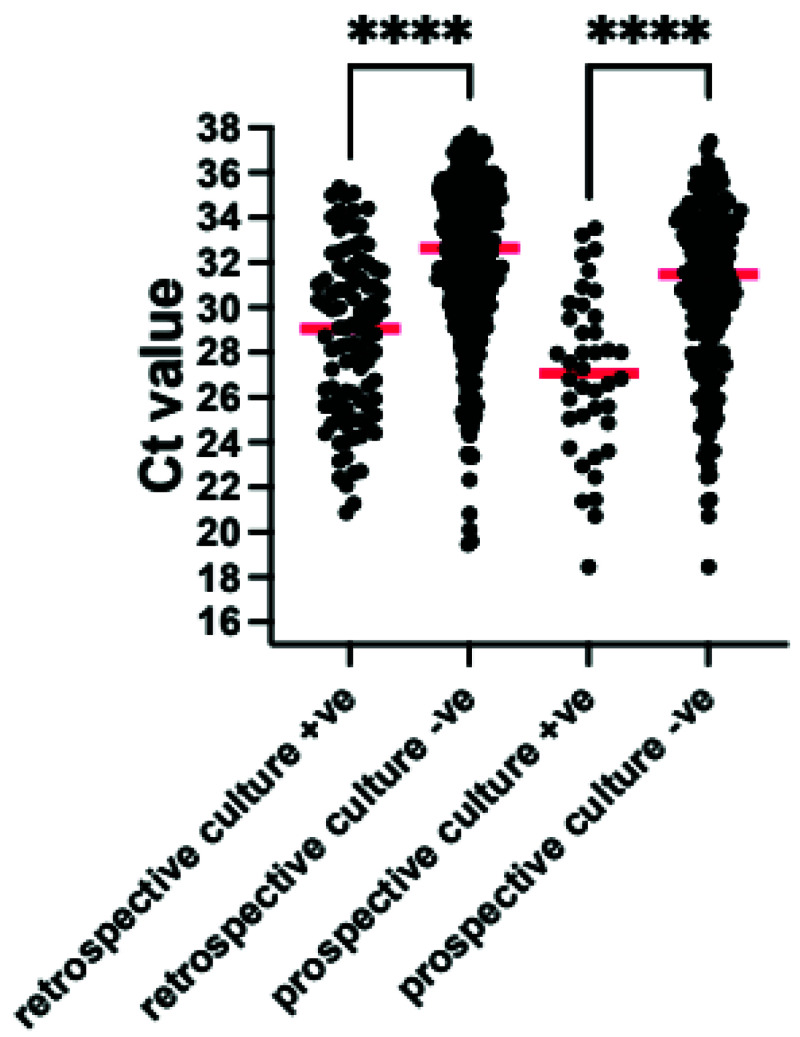
Comparison of Cycles to threshold (Ct) for bacteria and fungi detected by culture and TAC (culture +ve) and those which were detected by TAC alone (culture -ve) for both the VAP-RAPID VR (stored) and prospective evaluations. Red line indicates median value ****p<0.0001 by Mann-Whitney U test. Organisms which are not detected by standard culture techniques were excluded.

The card was tested against the stored samples available from the VAPrapid study ^15^. 128 samples with semi-quantitative culture results were available for analysis. 57 organisms were grown at or above 10 ^4^ colony forming units (CFU)/ml ^24, 32^, with 55 detected by TAC ( Table 2). The TAC detected a further 295 organisms, including 64 viruses and one atypical organism which the recruiting centres did not test for. Excluding tests for organisms not detectable by culture, 3425 tests on TAC were negative. Sensitivity was 97% (95% confidence interval (CI) 88-100%) and specificity 94% (95% CI 93-95%) ( Table 3). Organisms detected by both TAC and culture had a median cycles to threshold (Ct) value on the TAC of 29 (interquartile range (IQR) 26–32 range 20–35) whilst culturable organisms detected on TAC but not on culture had a median Ct value of 33 (IQR 30–35 range 20–40) ( Figure 2).

**Table 2.** Culture of microorganisms from 128 stored samples from the VAPrapid clinical trial ^15^ and results from the TAC. (CFU: colony forming units/ml, Ct: cycles to crossing threshold.).

Organism detected	Frequency of growth (≥10^4 CFU/ml) on conventional culture	Frequency by TAC (numbers detected at Ct≤32 shown in brackets)
**Gram negative**		
*Acinetobacter baumannii complex*	2	4 (3)
*Enterobacter aerogenes*	1	0 *
*Enterobacter cloacae*	2	7 (5)
*Enterococcus faecalis*	0	2 (1)
*Enterococcus faecium*	2	15 (9)
*Escherichia coli*	6	44 (16)
*Enterobacteriaceae* (not further specified)	0	7 (3)
*Haemophilus influenzae*	3	23 (19)
*Haemophilus haemolyticus*	1	0 *
*Klebsiella pneumoniae*	2	13 (6)
*Legionella* spp. (non-pneumophilia)	0	1 (1)
*Morexella catharralis*	1	5 (4)
*Morganella morganii*	0	1 (1)
*Mycoplasma pneumoniae*	0	1 (1)
*Neisseria meningitidis*	0	1 (1)
*Proteus* spp. ^#^	2	7 (5)
*Pseudomonas aeruginosa*	5	10 (9)
*Serratia marcescens*	1	5 (3)
**Gram positive**		
*Staphylococcus aureus*	21	32 (28)
*Staphylococcus epidermidis*	0	12 (3)
Other coagulase negative *Staphylococcus*	0	9 (1)
*Stenotrophomonas maltophilia*	2	11 (5)
*Streptococcus pneumoniae*	0	15 (10)
*Streptococcus pyogenes*	0	1 (1)
*Streptococcus* spp. (not further specified)	0	37 (25)
**Fungi**		
*Aspergillus fumigatus.*	0	1 (1)
*Candida albicans*	4	17 (7)
*Candida spp.*	2	5 (3)
**Viruses** (not tested for by conventional microbiology)		
Coronavirus OC43		1 (1)
Cytomegalovirus		6 (1)
Epstein-Barr Virus		15 (3)
Herpes simplex virus		34 (26)
Influenza A		3 (3)
Parainfluenza virus		1 (1)
Rhinovirus		4 (4)

*not on card ^#^ culture reported as *Proteus mirabilus*, on TAC reported as genus-level *Proteus spp.*

**Table 3.** 2x2 table for TAC vs culture for the retrospective stored sample study. Results presented for the 29 tests on the TAC covering culturable organisms (atypical bacteria, viruses and Pneumocystis jirovecii excluded).

	Reference standard (microbiological culture)		total
TAC	+ve	-ve	
+ve	55	230	285
-ve	2	3425	3423
total	57	3655	3712

### Prospective evaluation

**Figure 3.**
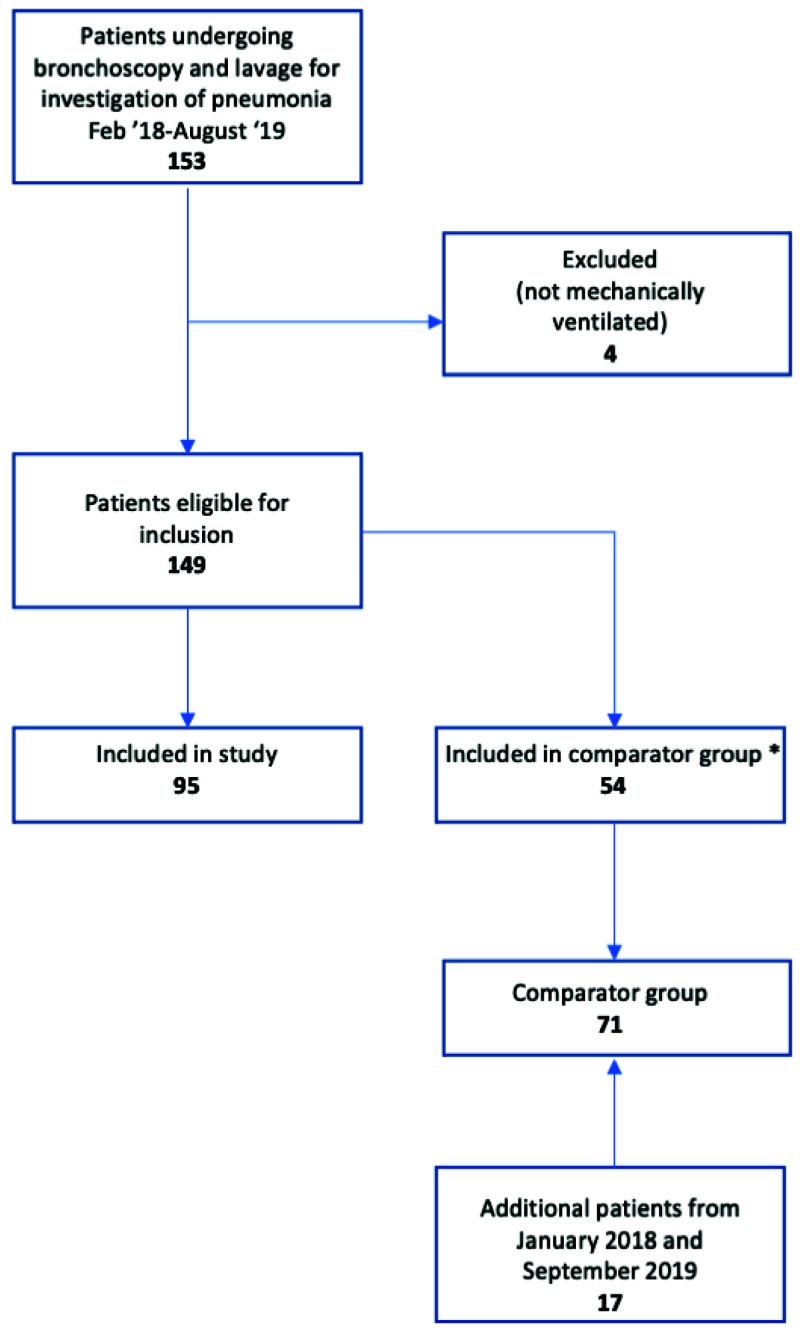
Study flow diagram.

**Figure 4.**
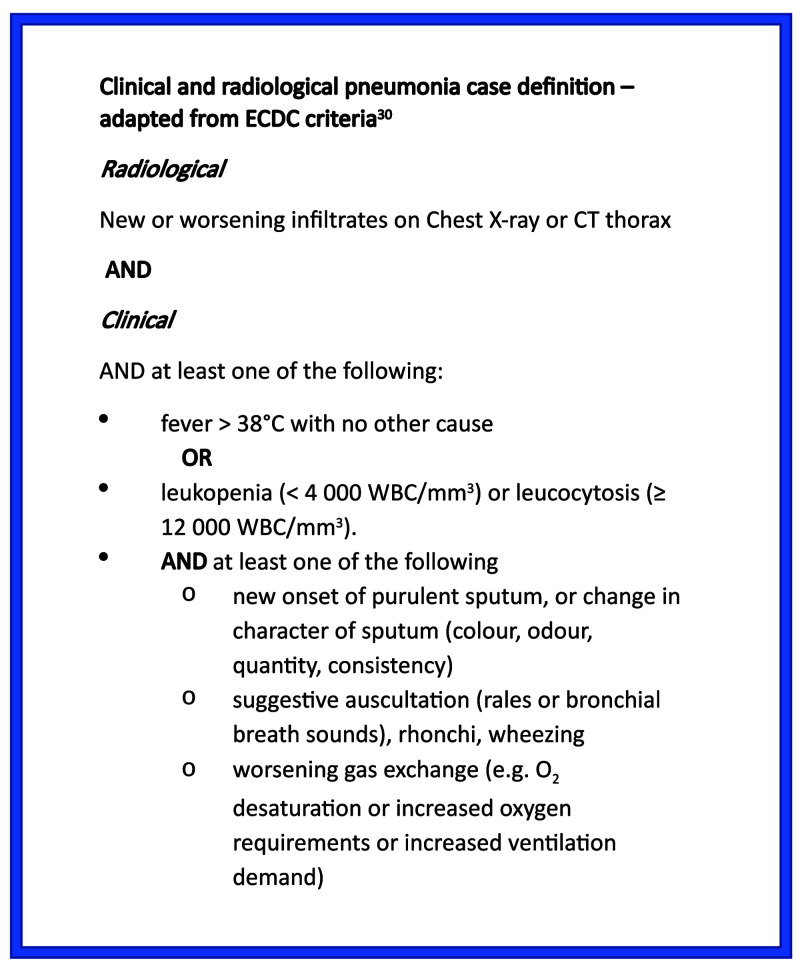
Clinical and radiological definition of pneumonia.

Between January 2018 and September 2019, 166 ventilated patients were investigated for pneumonia by bronchoscopy, and 95 were tested by TAC. No proxy decision maker approached refused consent, and 24 patients who regained capacity whilst in hospital were approached and all gave retrospective consent. Five patients were tested twice by TAC, having suffered a subsequent respiratory deterioration, so in total 100 TACs were run. 71 patients formed the comparator group ( Figure 3). Although inclusion criteria were pragmatic and only required senior clinician suspicion of pneumonia, 92% of cases met full ECDC criteria for clinical pneumonia ( Figure 4). Of the eight cases not meeting full ECDC criteria, one lacked a formal radiological report of infiltrates, one had no clinical signs of pneumonia, five had no signs of systemic inflammation and one patient lacked both radiological and systemic inflammation. Table 4 shows participant characteristics of the study population and comparator group.

**Table 4.** Baseline characteristics of study population. APACHE II, acute physiology and chronic health evaluation II, FiO _2_, fraction of inspired oxygen. (IQR: interquartile range).

Parameter	TAC patients (95 patients)	Comparator group (71 patients)
Median age (range)	60 (21–86)	62 (18–83)
n (%) female	41 (43 %)	27 (38 %)
Median (IQR) functional comorbidity index	1 (2)	1 (2)
n (%) with community-acquired pneumonia n (%) hospital-acquired pneumonia of HAP n (%) ventilator-associated	34 (36%) 61 (64%) 24 (39%)	20 (28%) 51 (72%) 27 (52%)
Median (IQR) APACHE II score on admission	16 (10)	16 (9)
% receiving antibiotics at time of lavage	82%	96%
Median (IQR) FiO _2_ prior to bronchoscopy	0.5 (0.25)	0.5 (0.30)
Median (IQR) white cell count (x10 ^9^/L)	10.5 (12.4)	10.7 (9.5)
Median (IQR) neutrophil count (x10 ^9^/L)	8.6 (11.5)	8.8 (8.63)
Median (IQR) C-reactive protein concentration (mg/L)	198 (153)	146 (154)
28-day mortality n (%)	30 (32%)	21 (30%)

**Figure 5.**
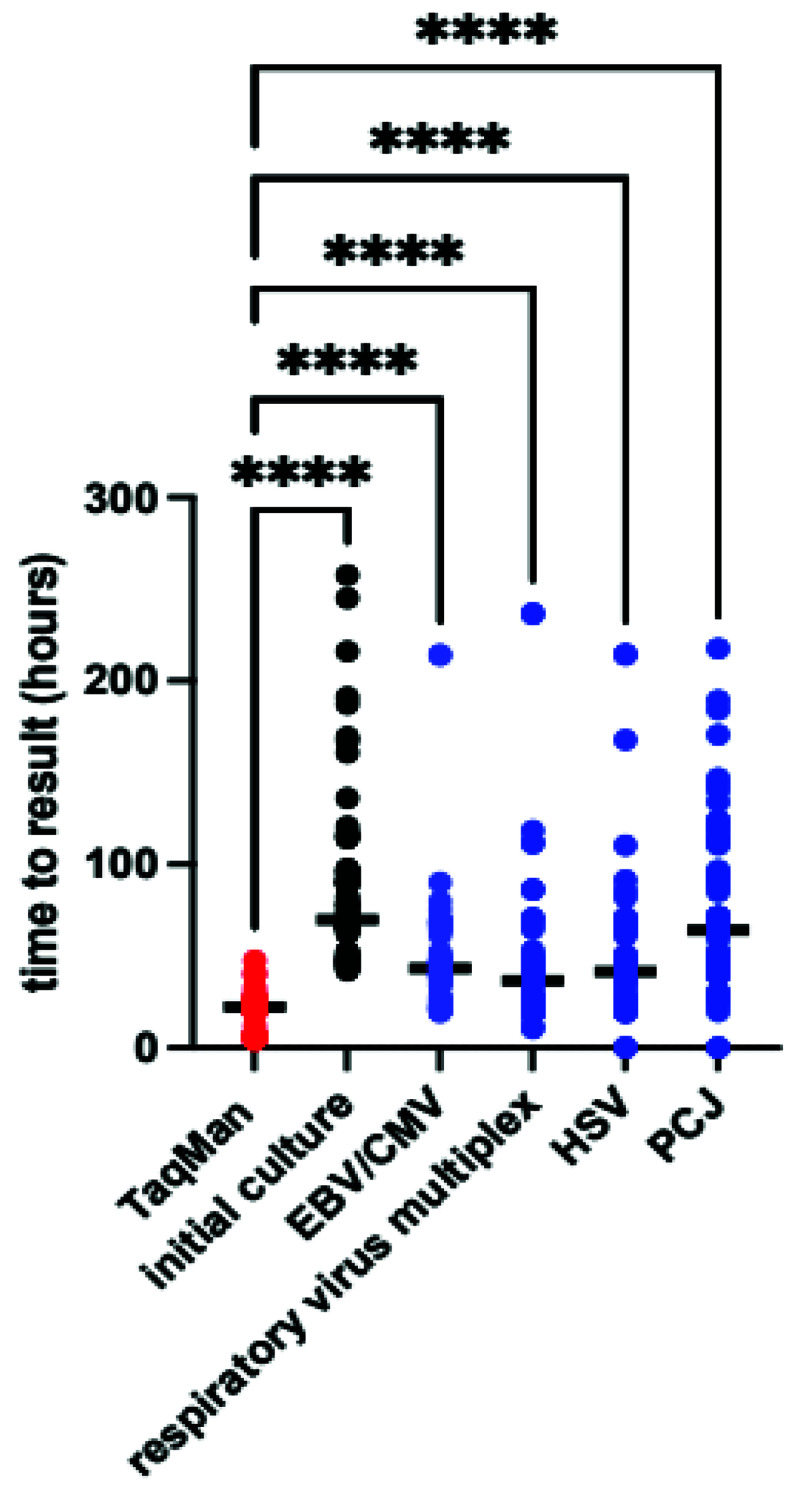
Time to result for TAC, conventional culture and conventional polymerase chain reactions. Bold line indicates median value. Median difference in test results vs TAC was 51hrs (IQR 41-69) for culture, 21 (IQR 16-40) for Epstein-Barr virus/cytomegalovirus(EBV/CMV), 16 (IQR 5-21) for respiratory virus multiplex, 19 (IQR 15-25) for Herpes Simplex Virus (HSV) and 42 (IQR 22-70) for *Pneumocystis jirovecii* (PCJ). P<0.001 by Kruskal-Wallis, **** p<0.001 by Dunn’s post-hoc test.

***Time to result*.** The median difference in time to result between TAC and conventional culture was 51 hours (IQR 41–69 hours p<0.0001 by Wilcoxon matched pairs), the TAC also returned results more rapidly than conventional PCR in almost all cases ( Figure 5). The minimum TAC time to return was 4 hours, with median time to result 22 hours (IQR 7–24 hours), most of the delays arose from samples taken outside routine working hours, whilst additional delays with conventional PCR results largely reflect laboratory workflow and batching of samples.

***Comparison of organisms detected by TAC compared to conventional microbiology*.** 178 organisms were identified from 100 samples on the TAC ( Table 5). Details of individual sample results are available as extended data ^33^. Conventional microbiology detected 66 organisms, with 61 detected by TAC. 27 patients had failure of internal control for one or more conventional PCR assays, covering 93 organisms. There were no TAC internal control failures and none of the organisms covered by the failed assays were detected on TAC or sequencing ( Table 5). Sensitivity and specificity were 92% (95% CI 83-98%) and 97% (95% CI 97-98%) respectively ( Table 6). Including failed and absent reference standards as ‘negative’ had minimal effect on diagnostic performance ( Table 7).

**Table 5.** Summary of organisms detected by conventional microbiological testing (left hand column), by TAC (middle column), and by microbial sequencing (right hand column).

Organism detected	Frequency (by conventional microbiology)	Frequency (by TAC)	Frequency (by sequencing)
**Bacteria**			
*Citrobacter freundii*	1 *	0	1 *
*Enterobacter cloacae*	2	8	3
*Enterococcus faecalis*	0	3	0
*Enterococcus faecium*	3	7	7
*Escherichia coli*	6	14	6
*Klebsiella pneumoniae*	3	5	3
*Enterobacteriaceae* (not further specified)	0	1	2
*Haemophilus influenzae*	0	3	2
*Legionella pneumophilia*	1 **	1	1
*Legionella* spp. (non-pneumophilia)	0	2	2
*Morexella catharralis*	0	1	0
*Mycoplasma pneumoniae*	0	1	1
*Proteus* spp.	0	2	0
*Pseudomonas aeruginosa*	2	5	4
*Serratia marcescens*	1	3	0
*Staphylococcus aureus*	2	8	8
*Staphylococcus epidermidis*	0	9	2
Other coagulase negative *Staphylococcus*	0	1	2
Other *Staphylococcus* spp. (not further specified)	0	0	1
*Stenotrophomonas maltophilia*	2	4	2
*Streptococcus pneumoniae*	1	9	6
*Streptococcus pyogenes*	0	1	0
*Streptococcus* spp. (not further specified)	0	23	10
Mixed upper respiratory tract flora	1	N/A	N/A
**Fungi**			
*Aspergillus spp.*	0 ***	1	0
*Candida albicans*	1	12	10
*Candida spp.*	0	1	1
*Pneumocystis jirovecii*	4	4	3
**Viruses**			
Coronavirus#	0	3	1
Cytomegalovirus	5	7	4
Epstein-Barr Virus	1	6	1
Herpes simplex virus	7	11	7
Human metapneumovirus	1	1	1
Influenza A	7	7	5
Influenza B	3	3	2
Parainfluenza virus	4	4	4
Rhinovirus	8	8	7

* One hit not found in same patient; not on card. **Legionella urinary antigen test positive. *** Positive bronchoalveolar lavage galactomannan enzyme immunoassay (>0.5 units) with CT consistent with fungal pneumonia and known risk factors but fungal cultures were not positive. # refers to human coronavirade OC43, 229E and NL63, no tests were undertaken for SARS-CoV2 and final testing occurred in August 2019.

**Table 6.** 2x2 table for TAC vs conventional microbiology for prospective study patients. Results presented for the 43 organisms routinely tested for in the laboratory, excluding 93 tests for patients where conventional PCR failed internal controls.

	Reference standard (conventional culture/PCR)		totals
TAC	+ve	-ve	
+ve	61	111	172
-ve	5	4030	4929
totals	66	4141	4207

**Table 7.** 2x2 table for TAC vs conventional microbiology for prospective study patients. Results presented for all 52 organisms tested on the card, with missing standard tests due to conventional PCR assay failure and non-testing treated as ‘negative tests’. Sensitivity and specificity were 92% (95% CI 83-98%) and 98% (95% CI 97-98%).

	Reference standard (conventional culture/PCR)		totals
TAC	+ve	-ve	
+ve	61	117	178
-ve	5	5017	5022
Totals	66	5134	5200

***Comparison by sequencing*.** 98 samples were available for sequencing. Metagenomic sequencing revealed 107 organisms, 100 of which were also detected by TAC ( Table 5 and extended data Table 1 ^33^).

Concerning the 10 organisms detected by conventional microbiology or sequencing but missed by TAC, one organism, that was positive by both culture and sequencing albeit in different patients, was *Citrobacter freundii*, for which we did not have a sequence on the card. A further five pathogens were detected by sequencing ( *Staphylococcus aureus, Legionella* spp. *,* and *Staphylococcus epidermidis)* or both culture and sequencing (two *E. faecium*). Although these five were detected by TAC, they did not pass the internal quality control standards required for reporting and were considered ‘negative’ results. The remaining three organisms, two rhinovirus by conventional PCR and one *Staphylococcus* spp. by sequencing, were not detected by TAC at all.

One case of *Aspergillus fumigatus* was detected on the TAC, and although no moulds were cultured, the lavage galactomannan antigen test was highly positive (5.92 optical density index (ODI), laboratory reference range <0.5 ODI).

***Quantitation*.** Twenty-five organisms were grown on conventional culture at ≥10 ^4^ CFU/ml, the conventional cut off for quantitative culture of lavage ^24, 32^. The median Ct for these organisms on the TAC was 27 (IQR 24-29, range 20–33). In contrast, culturable organisms detected on TAC but not on culture, and therefore likely to be present in lower concentrations and not reported for patients managed without TAC, had a median Ct of 32 (IQR 30-34, range 22–38) ( Figure 2).

**Figure 6.**
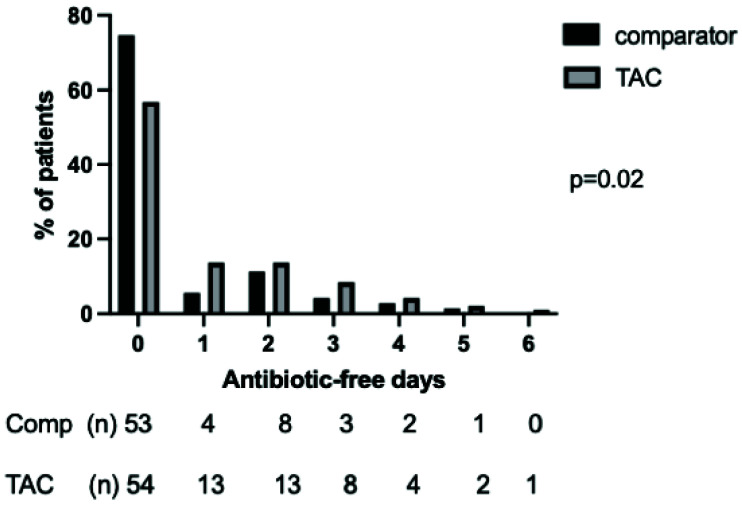
Distribution of days alive and free of antibiotics in the seven days following bronchoscopy and lavage in the TAC and comparator cohorts. Following first lavage only for patients who had more than one bronchoalveolar lavage during ICU admission. Numbers in each category and percentage shown below graph, p value by Mann-Whitney U test.

**Figure 7.**
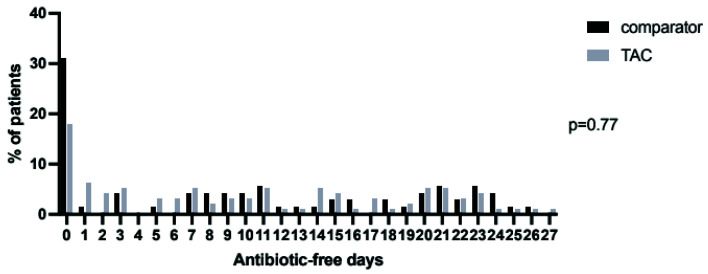
Distribution of days alive and free of antibiotics in the twenty eight days following bronchoscopy and lavage in the TAC and comparator cohorts. Following first lavage only for patients who had more than one bronchoalveolar lavage during ICU admission. P-value by Mann-Whitney U test.

***Antibiotic prescribing*.** Patients in the TAC and comparator cohorts had similar severity of illness, severity of respiratory failure and demographic features ( Table 4). Patients managed with the TAC had a significantly different distribution of AFDs to the comparator group in the seven days following bronchoscopy (p=0.02 by Mann-Whitney U-test), with more AFDs in the TAC cohort ( Figure 6). This difference did not retain significance over 28 days ( Figure 7). Overall 72 (76%) of TAC patients had their antibiotics changed in the seven days following bronchoscopy, with a total of 116 changes made ( Table 8). In the comparator group 50 (70%) of patients experienced a total of 65 changes. Whilst 63% of decisions in the TAC group led to de-escalation, only 37% of decisions in the comparator group were de-escalation decisions (OR 2.9 (95% CI 1.5-5.5) p=0.008 by Chi-squared). Decisions which were judged to be related to the TAC result were weighted further towards de-escalation (73% of all TAC-related changes, Table 8). 11 (30%) of escalations in the TAC group were judged to have been targeted escalations in response to TAC results. In a further six cases negative TAC results prompted investigation for alternative diagnoses.

**Table 8.** Detail of changes in antibiotic therapy in the seven days following lavage in the TAC and comparator cohorts. Changes judged to be TAC-related are shown in the left-hand sub-column for the TAC group. Several patients had more than one change in antibiotic therapy. *includes two de-escalations to prophylactic dose, ** includes two escalations from prophylactic to therapeutic dose.

Antibiotic change	Details of change	TAC cohort		Comparator cohort
		TAC-related	TAC-unrelated	
**De-escalation**	Stopping macrolides	14	3	6
	Stopping carbapenem or anti-pseudomonal penicillin	10	14	10
	Narrowing from carbapenem/antipseudomonal penicillin to narrower spectrum penicillin	7	0	1
	Stopping cotrimoxazole	6 *	1	0
	Stopping antivirals	3	0	0
	Stopping aminoglycosides	2	3	0
	Stopping other agents	6	4	7
**Escalation**	Start antivirals	7	0	1
	Start or broaden antifungals cover	3	7 **	6
	Broadened Gram negative cover (add anti-pseudomonal penicillin, aminoglycoside or carbapenem)	3	16	28
	Add glycopeptide	4	1	5
	Add cover for atypical organism	1	1	1

## Discussion

We demonstrate that a customised molecular diagnostic, designed to meet the needs of a specific clinical setting, produced accurate results in a clinically important time-frame and was associated with an increase in antibiotic-free days relative to the comparator group in the week following investigation. Diagnostic performance was similar when assessed in stored samples from multiple centres, implying a generalisable result.

Molecular diagnostic platforms for respiratory infection syndromes have, until recently, largely focussed on viral pathogens ^16^. However, the need to optimise antimicrobial therapy whilst limiting the over-use of these drugs has led to repeated calls for bacterial-focussed diagnostics ^16, 34^. TACs have been previously reported for use in pneumonia ^19, 35, 36^. However, apart from our previous report ^19^ that demonstrated limited clinical impact due to restricted organism coverage, none of the other reports have included ventilated patients and were restricted to retrospective analysis of stored samples. Commercial multiple-pathogen arrays that include respiratory bacteria have recently become available, however most reports of their use in ventilated patients remain limited to describing diagnostic performance, and reporting ‘potential’ to change antimicrobial therapy rather than impact on clinical practice ^3, 37, 38^. Concerns have been raised about the risks of over-treatment from molecular diagnostics ^16, 17, 39, 40^, whilst conversely promising tests with the potential to change therapy have not always proven this in clinical practice ^15, 19^. These commercially available assays lack the broad coverage and customisability of the TAC, with consistent concerns raised around limited organism coverage adversely impacting treatment decisions ^3, 17, 19, 37, 38^.

**Figure 8.**
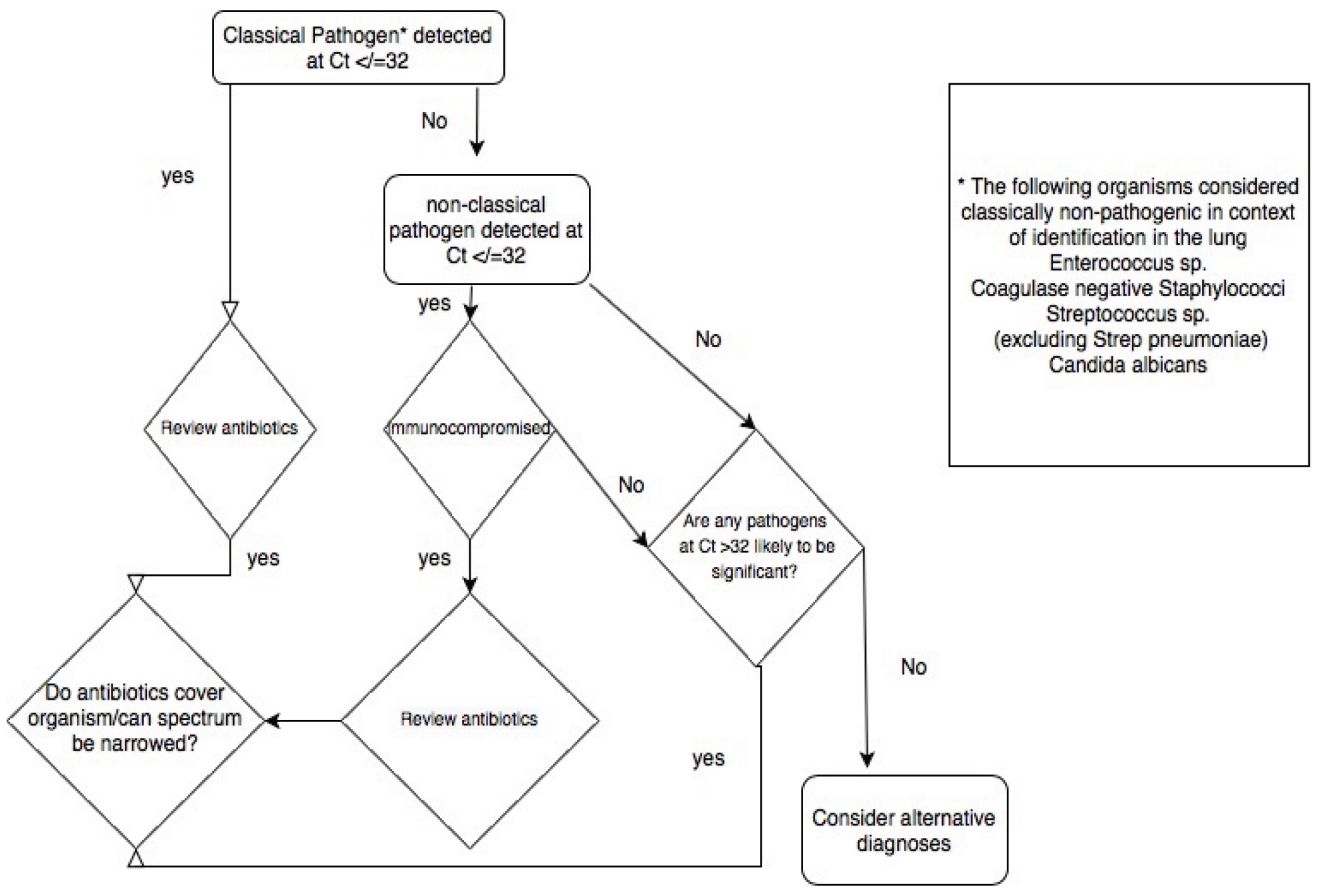
Schematic representation of the proposed clinical decision tree arising from the results of the TaqMan array.

There is now widespread acceptance of the presence of a respiratory microbiome ^41, 42^ and the lungs of ventilated patients present a challenge to highly sensitive molecular diagnostics ^16, 17^. The proximal respiratory tract of ventilated patients becomes rapidly colonised with predominantly Gram negative organisms ^43, 44^. This can occur in the absence of infection, and there is a risk that highly sensitive techniques will detect colonising organisms, driving unintended increases in antimicrobial use ^16^. The use of protected lower airway specimens, with growth ≥10 ^4^ CFU/ml for BAL have been used to distinguish infection from colonisation ^22, 32, 45^. We adapted this approach in this study, using the quasi-quantitative Ct value provided by RT-PCR and testing protected bronchoalveolar samples. Using the comparison of the Ct values of organisms detected by culture and those detected by TAC without culture, we suggest that a Ct threshold of 32 be used to suggest infecting rather than colonising organism ( Figure 2 and Figure 8).

One of the problems that has beset bacterial diagnostics studies has been the absence of a ‘gold standard’ against which the candidate can be assessed ^16, 34, 46^, as conventional culture is imperfect. For this study we supplemented comparison against conventional microbiology (culture and viral PCR) with metagenomic sequencing. 10 organisms identified by conventional microbiology or sequencing were not detected by the TAC. Overall, the TAC detected more organisms than either culture or sequencing, reflecting the higher sensitivity of qPCR. However, without a perfect validation method we cannot be certain these were not ‘false positives’ and have counted them as such for the calculation of specificity. The sequencing and culture results give clinicians considerable confidence in the results provided.

The selection of organisms targeted on the card was crucial, and informed by our previous experience where omission of key organisms significantly limited the impact of a similar card ^19^. Given the case mix of our unit, with a high proportion of immunosuppressed patients, we opted to include a number of low pathogenicity organisms, (i.e. coagulase-negative *Staphylococci* (CNS), *Enterococci* and *Candida albicans*), as well as Herpesviridae, which were routinely tested for prior to this study. The detection of these organisms can be challenging to interpret ^47^, given that many critically ill patients have a degree of immunoparesis, even if not classically immunosuppressed ^6, 48^, and so their significance remains uncertain. As our laboratory routinely reported these organisms on conventional microbiology the clinical team were already confronted with this issue.

It is increasingly apparent that the microbiome of the lungs of ventilated patients undergoes a shift, with increasing dominance of enteric organisms which may enter the lungs via gut translocation and microaspiration ^49, 50^. Determining whether enteric organisms, even those such as *E. coli* which are conventionally considered pathogenic in the lung, are the cause or the consequence of pulmonary inflammation remains a challenge. However this is a challenge which besets both conventional and molecular diagnostics, and one which is likely to take further developments in host-response diagnostics to resolve ^10, 13, 15, 41, 44^

The inclusion of CNS aids with the interpretation of the detection of the mecA gene, which is commonly carried by these organisms, thus helping identify MRSA. The lack of CNS sequences on commercial cards has been noted to impair interpretation of mecA detection on other molecular diagnostic platforms ^37, 51^. The ready customisability of the TAC would allow units to remove such organisms, as well as add other organisms that emerge as a threat as we have done subsequently during the COVID-19 pandemic ^52^. Although we undertook the steps noted in the methods section to validate the TAC prior to use, the range of organisms encountered in the retrospective and prospective cohorts’ clinical samples did not cover the full range of organisms on the TAC. Some organisms not seen in this report had been detected in previous studies using the same assays in an earlier iteration of the card ^18, 19^. We must therefore be somewhat cautious about the interpretation of the assays without clinical sample validation, and continue to monitor card performance as experience with this card develops in our centre.

The use of a contemporaneous comparator cohort allowed for comparisons of antibiotic prescribing within the context of the implementation of the TAC and any heightened awareness of antimicrobial stewardship it may have engendered. Despite this, the comparator cohort saw a greater proportion of escalation decisions in the week following lavage, and had fewer antibiotic-free days. The 28 day mortality was similar between the two groups, and both had similar duration of ventilation and ICU length of stay ( Table 4), which suggests that the change in antibiotic therapy was not associated with harm. The lack of difference in AFDs at day 28 is unsurprising, as suspected pneumonia is only one of multiple drivers of antibiotic use. Although the comparator and TAC groups had similar characteristics, our observational design means that we cannot be certain that unmeasured confounders did not contribute to the effects seen. Replication in additional settings with distinct approaches to stewardship is required before we can be certain of its external generalisability, whilst evaluation in a randomised, controlled trial would help reduce any bias that may have arisen from our observational study design.

### Implementation of TaqMan array

Our experience leads us to suggest the following approach to using the TAC. Where a pathogenic organism(s) is detected at Ct value of ≤32, antimicrobials can be adjusted to target the organism(s) detected, in light of known local resistance patterns and the patient's history carriage of antimicrobial-resistant organisms. Pathogenic organisms detected at a Ct of >32 are likely to be colonisers or contaminants, although detection of respiratory viruses or obligate pathogens such as *Legionella spp* or *Mycoplasma pneumoniae* at higher Ct values remains significant. The detection of low pathogenicity organisms needs to be interpreted in light of the patient’s known or suspected immune status. Among the immunocompromised, high levels of such organisms, especially if it is the sole pathogen detected, may prompt treatment. In patients in whom no relevant pathogens are detected (i.e. all organisms are at low levels, low pathogenicity organisms are detected in immunocompetent hosts, or no organisms are detected at all) consideration should be given to alternative sites of infection, alternative diagnoses and where clinical suspicion of infection is low, stopping antibiotics ( Figure 8). We used the TAC in the context of an existing daily microbiology-ICU multi-disciplinary team meeting, and as noted above generalisability of our findings to other contexts will require replication in those settings. We note that further clinical sample validation is required for rare organisms not encountered in our retrospective and prospective cohorts. Confirmation of safety of TAC-driven changes in antimicrobials will be best established in prospective randomised trials.

## Conclusions

This study established a molecular diagnostic test to meet the needs of a particular intensive care unit, although generalisability was demonstrated through testing samples from multiple other units. The strength of the TAC is that it allows customisation and rapid modification to address emerging threats and ensure broad coverage. Implementation in the context of an antimicrobial stewardship program led to significant impact on antimicrobial prescribing. We believe this approach represents a promising new approach to the management of severe pneumonia but this requires testing in the context of well-designed randomised clinical trials.

## Data Availability

European Nucleotide Archive (ENA): Bronchialveolar_lavage_metagenomics (Project PRJEB29011). Accession number ERP111277; https://identifiers.org/ena.embl:ERP111277 ENA: BAL_metagenomics__viral_enrichment (Project: PRJEB29014). Accession number: ERP111280; https://identifiers.org/ena.embl:ERP111280 ENA: Bronchialveolar_lavage_microbiome (Project: PRJEB29919). Accession number: ERP112277; https://identifiers.org/ena.embl:ERP112277 ENA: ICU_metagenomics_Using_16S_rRNA_analysis_for_assessing_the_respiratory_bacterial_infection_threat_to_immunocompromised_patients_within_Intensive_Care_Units (Project: PRJEB16762). Accession number: ERP018622; https://identifiers.org/ena.embl:ERP018622 Patient data is not publicly available for confidentiality reasons in line with the approved study protocol, but anonymised data can be obtained through contact with the corresponding author, subject to appropriate data sharing agreements being in place. Data sharing agreements will be arranged by Cambridge University Hospitals NHS Foundation Trust, as the study sponsor. Zenodo: Rapid Pathogen Identification in Ventilated Patients with Pneumonia. https://doi.org/10.5281/zenodo.5081880 ^20^ This project contains the following data: TAC protocol.docx (study protocol) Zenodo: Use of antibiotics in ICU patients undergoing lavage-based diagnosis of pneumonia -protocol for retrospective study https://doi.org/10.5281/zenodo.5519073 ^53^ This project contains the following data anonymised data BAL protocol 1point 1 5 2 20.pdf Zenodo: TaqMan array sequence set for 52-target respiratory pathogen array. https://doi.org/10.5281/zenodo.5519133 ^23^ This project contains the following data VAP 96 TAC card sequence file.pdf Zenodo: Extended data table 1: Taqman array card results showing all individual target hits with Ct values and whether validated by conventional microbiology and/or microbial sequencing. https://doi.org/10.5281/zenodo.5519136 ^33^ This project contains the following data Extended data table 1.pdf Zenodo: STARD checklist for ‘Development and implementation of a customised rapid syndromic diagnostic test for severe pneumonia’. https://doi.org/10.5281/zenodo.5081937 ^54^ Data are available under the terms of the Creative Commons Attribution 4.0 International license (CC-BY 4.0).
