# Supplementary material for: Development and implementation of a customised rapid syndromic diagnostic test for severe pneumonia
Source: Wellcome Open Res. 2022 Oct 12;6:256. Originally published 2021 Oct 11. [Version 3] doi: 10.12688/wellcomeopenres.17099.3 (PMC9617073; doi:10.12688/wellcomeopenres.17099.3)
Supplement: Peer review history

# Peer review: Reviewer response for version 3

**DOI:** 10.21956/wellcomeopenres.20471.r52795

**Published:** 2022-10-28

**Authors:** Bastiaan Haak

**Affiliations:** 1 Center for Experimental and Molecular Medicine, Amsterdam Infection & Immunity Institute, Amsterdam UMC, location AMC, University of Amsterdam, Amsterdam, The Netherlands

The authors have addressed my comments appropriately.

Is the rationale for developing the new method (or application) clearly explained?

Yes

Is the description of the method technically sound?

Yes

Are the conclusions about the method and its performance adequately supported by the findings presented in the article?

Yes

If any results are presented, are all the source data underlying the results available to ensure full reproducibility?

Yes

Are sufficient details provided to allow replication of the method development and its use by others?

Yes

Reviewer Expertise:

Infectious disease, (lung) microbiome

I confirm that I have read this submission and believe that I have an appropriate level of expertise to confirm that it is of an acceptable scientific standard.

# Peer review: Reviewer response for version 2

**DOI:** 10.21956/wellcomeopenres.19815.r51054

**Published:** 2022-06-29

**Authors:** Bastiaan Haak

**Affiliations:** 1 Center for Experimental and Molecular Medicine, Amsterdam Infection & Immunity Institute, Amsterdam UMC, location AMC, University of Amsterdam, Amsterdam, The Netherlands

The authors present an interesting manuscript in which they describe the potential of an agnostic molecular diagnostic approach (TAC) for direct detection of emerging pathogens from clinical specimens in patients with pneumonia. They show that their approach yields highly sensitive results in significantly shorter time as compared to time required for culture-dependent assays. In addition, they demonstrate that the use of TAC led to a significant reduction in antibiotic free days, which could be of importance in improving antibiotic stewardship practices. Overall, the manuscript is well written and the methodological steps are sound. This manuscript has been reviewed extensively prior to my review, and I do not have much to add. 

 However, I agree with reviewer 1 that certain pitfalls will have to be addressed before such methods can be used as part of widespread clinical practice. A specific point of concern is the use of TAC on broncho-alveolar lavage samples leading to the detection of high amounts of bacterial communities originating from the gastrointestinal tract (i.e. *Escherichia coli and Enterococcus spp.*). It is unclear to what extent these bacteria contribute to clinical disease, as recent studies have shown that it is likely that these microbial communities translocate from the gut into the lower airways following the development of a respiratory infection. Therefore, identification of these bacteria complicates rational antibiotic selection, as it is unclear if these bacteria are the cause or the effect of the disease. This point has been raised by reviewer 1 in the context of the high detection rates of *Candida *in the TAC cohort. However, I would request the authors to write an additional paragraph in which these specific limitations are addressed.

Is the rationale for developing the new method (or application) clearly explained?

Yes

Is the description of the method technically sound?

Yes

Are the conclusions about the method and its performance adequately supported by the findings presented in the article?

Yes

If any results are presented, are all the source data underlying the results available to ensure full reproducibility?

Yes

Are sufficient details provided to allow replication of the method development and its use by others?

Yes

Reviewer Expertise:

Infectious disease, (lung) microbiome

I confirm that I have read this submission and believe that I have an appropriate level of expertise to confirm that it is of an acceptable scientific standard, however I have significant reservations, as outlined above.

## References

[1] : Enrichment of the lung microbiome with gut bacteria in sepsis and the acute respiratory distress syndrome. *Nature Microbiology* .2016;1(10) : 10.1038/nmicrobiol.2016.113 10.1038/nmicrobiol.2016.113 PMC507647227670109

[2] : Lung Microbiota Predict Clinical Outcomes in Critically Ill Patients. *American Journal of Respiratory and Critical Care Medicine* .2020;201(5) : 10.1164/rccm.201907-1487OC 555-563 10.1164/rccm.201907-1487OC PMC704746531973575

# Response

**Published:** 2022-10-10

**Authors:** Andrew Conway Morris

We thank the reviewer for their comments.  The reviewer is correct that distinguishing infecting organisms from organisms that colonise the respiratory tract is a challenge. It is  one which besets both conventional, culture-based microbial diagnostics as well as molecular diagnostics.  Furthermore it is unclear to what extent changes in the pulmonary microbiome contribute to the syndrome of pneumonia as it is recognised by clinicians.  This is not something that can be resolved by this study, but we have added the following section to the discussion to acknowledge the issues raised.

 “It is increasingly apparent that the microbiome of the lungs of ventilated patients undergoes a shift, with increasing dominance of enteric organisms which may enter the lungs via gut translocation and microaspiration ^46,47^.  Determining whether enteric organisms, even those such as E coli which are conventionally considered pathogenic in the lung, are the cause or the consequence of pulmonary inflammation remains a challenge. However this is a challenge which besets both conventional and molecular diagnostics, and one which is likely to take further developments in host-response diagnostics to resolve ^10,13,15,38,41^.”

# Peer review: Reviewer response for version 2

**DOI:** 10.21956/wellcomeopenres.19815.r50420

**Published:** 2022-06-14

**Authors:** Tim Felton

**Affiliations:** 1 Division of Infection, Immunity and Respiratory Medicine, The University of Manchester, Manchester, UK

I have no further comments. The authors have addressed my previous comments/questions appropriately.

Is the rationale for developing the new method (or application) clearly explained?

Yes

Is the description of the method technically sound?

Yes

Are the conclusions about the method and its performance adequately supported by the findings presented in the article?

No

If any results are presented, are all the source data underlying the results available to ensure full reproducibility?

Yes

Are sufficient details provided to allow replication of the method development and its use by others?

Yes

Reviewer Expertise:

Infection diagnostics, antibiotic pharmacology

I confirm that I have read this submission and believe that I have an appropriate level of expertise to confirm that it is of an acceptable scientific standard.

# Peer review: Reviewer response for version 1

**DOI:** 10.21956/wellcomeopenres.18886.r46380

**Published:** 2021-11-22

**Authors:** Tim Felton

**Affiliations:** 1 Division of Infection, Immunity and Respiratory Medicine, The University of Manchester, Manchester, UK

The manuscript describes preliminary evidence of a multiplex assay to identify respiratory tract pathogens in critically ill adults. The manuscript is well written and easy to read.

 Commercial multiplex PCR diagnostic assays would be expected to demonstrate the sensitivity and specificity of each of the individual assays. Whilst this is onerous given that some of the pathogens are uncommon, some of the PCR targets are not present at all in the validation cohort.

 The authors make no comment of safety related to changing antibiotic prescribing. Given that some of the targets are unvalidated and the PCR result may have resulted in antibiotics being withheld or stopped early it would be helpful to understand the outcome of patients.

 It is unclear to me how the TaqMan assay results in a reduction in antibiotics given the huge number of pathogens identified in patients. The authors state that the results were released into clinical practice without specific advice but presumably some advice was given as to which pathogens not to treat? Additionally the diagnostic accuracy analysis is a little misleading given how much better the new test performs compared to the gold standard. Is a conventional diagnostic accuracy analysis the best analysis to perform?

 A lot of Candida species was identified in the respiratory tract. Candida is generally considered a coloniser in the lung and not a cause of pneumonia. Did the authors treat each of the cases of candida? More detail of the management of candida in the lung is required – did these patients all receive antifungal therapy? If candida was regarded as a colonising organism it should be considered a negative result in the diagnostic accuracy analysis.

 Overall a really interesting manuscript. I think a number of issues need to be addressed before this assay can be rolled out into clinical practice: validation of the rare pathogens, patient safety, out performance of the gold standard. It would strengthen the manuscript if the authors added a section to the discussion to outline next steps. This would make it clearer that the assay is not ready for mass adoption (which is suggested).

Is the rationale for developing the new method (or application) clearly explained?

Yes

Is the description of the method technically sound?

Yes

Are the conclusions about the method and its performance adequately supported by the findings presented in the article?

No

If any results are presented, are all the source data underlying the results available to ensure full reproducibility?

Yes

Are sufficient details provided to allow replication of the method development and its use by others?

Yes

Reviewer Expertise:

Infection diagnostics, antibiotic pharmacology

I confirm that I have read this submission and believe that I have an appropriate level of expertise to confirm that it is of an acceptable scientific standard, however I have significant reservations, as outlined above.

# Response

**Published:** 2022-05-05

**Authors:** Andrew Conway Morris

We thank Dr Felton for his review and comments.  To address each in turn

**R1:** Commercial multiplex PCR diagnostic assays would be expected to demonstrate the sensitivity and specificity of each of the individual assays. Whilst this is onerous given that some of the pathogens are uncommon, some of the PCR targets are not present at all in the validation cohort.

**A1: **We acknowledge that whilst the samples tested in both the retrospective cohort validation and prospective evaluation contained a wide range of organisms they did not include all organisms for which we have sequences on the card.  Previous studies (Steensals D et al and Jones N  et al, references 18 and 19 in the manuscript) used many of the same assays used in this TAC, and identified some organisms not seen in our cohort.  As noted in the  methods, ‘technical validation section’, we did test the sequences on the cards against synthetic control plasmids to ensure their technical function, and also tested against extracts from known positive cultures in the lab.  For atypical organisms where we did not have positive cultures in the lab ( *C burnetti. C psittaci, C pneumonia *and *M pneumoniae*) commercially available controls were purchased from Vircell, Granada, Spain.  The only organism which was validated entirely by synthetic control plasmid was *Elizabethkingia meningoseptica*, which was a prevalent organism in our hospital 5 years ago but has not been detected on culture recently.  We agree that detection in clinical samples with confirmed culture of these organisms would be the gold standard.  With the card being used in Cambridge University Hospitals for adult pneumonia, and an ongoing study in paediatric pneumonia we have continued to accumulate data and review card performance regularly. Through this, some of the targets that were not validated in clinical samples this study have subsequently been validated. We have adjusted the methods to include further information regarding technical validation against clinical culture and commercial controls, and altered the discussion section to highlight this potential issue.

 The specific changes are methods “…….c overing all targets except *C burnetti. C psittaci, C pneumonia* *,* *M pneumoniae* and *Elizabethkingia meningoseptica*. For C burnetti. C psittaci, C pneumonia and M pneumoniae control DNA extracts were purchased (Vircell, Granada, Spain, Catalogue numbers MBC018, MBC013, MBC011 and MBC035 respectively). “

 Discussion “Although we undertook the steps noted in the methods section to validate the TAC prior to use, the range of organisms encountered in the retrospective and prospective cohorts’ clinical samples did not cover the full range of organisms on the TAC.  Some organisms not seen in this report had been detected in previous studies using the same assays in an earlier iteration of the card ^18,19^.  We must therefore be somewhat cautious about the interpretation of the assays without clinical sample validation, and continue to monitor card performance as experience with this card develops in our centre.”

**R2:**The authors make no comment of safety related to changing antibiotic prescribing. Given that some of the targets are unvalidated and the PCR result may have resulted in antibiotics being withheld or stopped early it would be helpful to understand the outcome of patients.

**A2:**We agree that safety is an important outcome, and note in the introduction that potential for harm may cut both ways (reference 4 summarising the evidence of antimicrobial harm, whilst reference 5 speaks to the risks of delayed and inappropriate therapy).  Although we reported mortality being similar between the comparator and prospective TAC evaluation groups (table 4) in the context of demographically similar groups, we have now included data showing similar duration of ICU stay and duration of ventilation.  We have added a note of these values in the discussion, and we noted previously that a randomised study would be the best way to address the potential of residual confounding. 

**R3:**It is unclear to me how the TaqMan assay results in a reduction in antibiotics given the huge number of pathogens identified in patients. The authors state that the results were released into clinical practice without specific advice but presumably some advice was given as to which pathogens not to treat? Additionally the diagnostic accuracy analysis is a little misleading given how much better the new test performs compared to the gold standard. Is a conventional diagnostic accuracy analysis the best analysis to perform?

**A3: **We examined the antibiotic therapy following lavage, and whilst we agree that the TAC did detect more organisms we also found that when changes occurred this tended to be towards de-escalation, although there were examples of targeted escalations as well.  Although we have looked  at decisions around the initiation or cessation of individual antibiotics and their relationship to the TAC, we acknowledge in the paper that this judgement is inherently subjective. This is why our primary analysis of antibiotic use is overall antibiotic-free days between the groups and change in antibiotics, which are taken from the drug prescriptions and thus do not require subjective judgement.  This is likely to under-estimate the impact of the TAC as some antibiotics may be prescribed for reasons other than suspected pneumonia. 

 In terms of microbiology advice, being in the study did not mandate a specific course of action.  Whilst results were released to the clinical team, our unit has a daily microbiology-ICU multi-disciplinary round where all microbiology results (including those from the TAC) are considered and decisions made around antimicrobial prescription.  This is noted in the methods, ‘return of results to the clinical team’ section.  In the discussion we noted “Replication in additional settings with distinct approaches to stewardship is required before we can be certain of its external generalisability”.  We have further emphasised this in the discussion section ‘implementation of the TaqMan array’ with an additional sentence “We used the TAC in the context of an existing daily microbiology-ICU multi-disciplinary team meeting, and as noted above generalisability of our findings to other contexts will require replication in those settings.”

 We agree that conventional measures of diagnostic accuracy are difficult in evaluation of tests with greater sensitivity than the existing clinical standard.  This does run the risk of understating the apparent specificity of the test, as we note in the discussion “Overall, the TAC detected more organisms than either culture or sequencing, reflecting the higher sensitivity of qPCR. However, without a perfect validation method we cannot be certain these were not ‘false positives’ and have counted them as such for the calculation of specificity”  however we felt that understating the apparent specificity and acknowledging this risk was the best approach.  Equally we acknowledge that culture may miss organisms that are not culturable, which is why we undertook the sequencing. The lack of a consensus standard on the interpretation of sequencing for clinical samples meant that we didn’t include these data as part of the primary outcome measure of sensitivity (which was vs conventional microbiology), but reported them separately.  However we think that the data from both conventional microbiology and sequencing are supportive of the diagnostic performance of the TAC.  We feel that presenting the summary diagnostic test performance as % sensitivity and % specificity is the most easily interpretable presentation, and is consistent with our pre-defined study protocol outcomes.

**R4:**A lot of Candida species was identified in the respiratory tract. Candida is generally considered a coloniser in the lung and not a cause of pneumonia. Did the authors treat each of the cases of candida? More detail of the management of candida in the lung is required – did these patients all receive antifungal therapy? If candida was regarded as a colonising organism it should be considered a negative result in the diagnostic accuracy analysis.

**A4:** We agree that candida in the lung are not normally considered a cause of pneumonia and in only two cases were anti-fungals started by the treating clinicians on the basis of detection of candida (table 8), one of these patients was immunocompromised and the other had an oesophageal perforation and both were considered to be at risk of systemic candidiasis. The third initiation of antifungals noted in table 3 was in response to the detection of aspergillus on the TAC.  Our diagnostic accuracy referred to the detection of organisms rather than the detection of pneumonia, so we compared all organisms detected on TAC to those detected by conventional microbiology without inferring a diagnosis of pneumonia.  Pneumonia remains a difficult diagnosis to make in ventilated patients and we think that detection or non-detection of organisms is a more objective measure.  We discuss our reasons for including ‘low pathogenicity organisms’ on the TAC in the discussion section. 

**R5:**Overall a really interesting manuscript. I think a number of issues need to be addressed before this assay can be rolled out into clinical practice: validation of the rare pathogens, patient safety, out performance of the gold standard. It would strengthen the manuscript if the authors added a section to the discussion to outline next steps. This would make it clearer that the assay is not ready for mass adoption (which is suggested).

**A5:** We thank Dr Felton for these comments.  As per A1 above, we agree that assays for rare organisms that have not been previously validated need to be validated in clinical samples alongside our culture and synthetic plasmid validation.  We also agree that testing at other sites, ideally in the context of a randomised controlled trial, would help address the questions of generalisability and safety of this approach. We have added further sentences to the discussion section, implementation of the TaqMan array and conclusions to emphasise these points.  These additional sentences are “We note that further clinical sample validation is required for rare organisms not encountered in our retrospective and prospective cohorts.  Confirmation of safety of TAC-driven changes in antimicrobials will be best established in prospective randomised trials” and “We believe this approach represents a promising new approach to the management of severe pneumonia but this requires testing in the context of well-designed randomised clinical trials **.”**
